# Spatial associations between neuronal membrane damage and vasculature after repetitive diffuse TBI in pigs

**DOI:** 10.1186/s40478-026-02309-8

**Published:** 2026-05-05

**Authors:** Kathryn L. Wofford, Erin M. Purvis Conway, Victor P. Acero, Jerry Gao, Olivia M. Rivellini, Emory Kuo, Constance J. Mietus, Kevin D. Browne, John C. O’Donnell, D. Kacy Cullen

**Affiliations:** 1 Department of Neurosurgery, Center for Brain Injury and Repair, University of Pennsylvania, Philadelphia, PA 19104 USA; 2 Center for Neurotrauma, Neurodegeneration and Restoration, Corporal Michael J. Crescenz VA Medical Center, Philadelphia, PA 19104 USA; 3 Department of Neuroscience, Perelman School of Medicine, University of Pennsylvania, Philadelphia, PA 19104 USA; 4 Department of Bioengineering, University of Pennsylvania, Philadelphia, PA 19104 USA

**Keywords:** Diffuse traumatic brain injury, Neuropathology, Vasculature, Membrane permeability, Injury biomechanics

## Abstract

Closed-head traumatic brain injury (TBI) generally results in diffusely distributed neuropathology. However, factors influencing the micro-scale distribution of this neuropathology remain unknown. Because neurovasculature exhibits different mechanical properties from the surrounding parenchyma, we postulated that these material transitions would increase the likelihood of biophysical damage and resultant neuropathology. Accordingly, we measured the incidence of neuronal plasmalemmal damage in perivascular domains using an established porcine model of single or repetitive closed-head rotational acceleration induced TBI. We found a significant increase in the proportion of regions containing permeabilized neurons in the cortex, thalamus, and midbrain following a single head rotation. We next employed an unbiased sampling methodology that mapped the distribution of vasculature (location, size, and directionality) and permeabilized neurons to determine if neuronal permeability increased based on proximity to vasculature. We identified an increase in the number of permeabilized neurons and a significant decrease in the number of larger (> 100 μm) vessels in the midbrain after repetitive TBI separated by 3 days. Furthermore, we identified that perivascular areas exhibited an increased density of neuronal permeability relative to more distal, extraperivascular spaces but only when excluding blood vessels that had no associations with permeabilized neurons. Neuronal permeability distribution was not affected by vessel size. These data suggest that vascular changes and neuronal damage may be unique consequences based on injury biomechanics, timing between repetitive injuries, and neuroanatomical features. While neuroanatomical features such as distance to the nearest vessel contribute to the distribution of neuronal mechanoporation, our findings indicate that perivascular proximity is a secondary, not primary, factor influencing this distribution. More research is needed to identify other factors that affect the susceptibility and distribution of neuronal plasmalemmal pathology.

**Graphical abstract:**

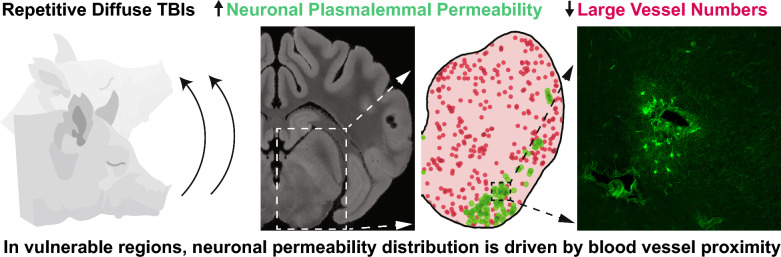

**Supplementary Information:**

The online version contains supplementary material available at 10.1186/s40478-026-02309-8.

## Introduction

Traumatic brain injury (TBI) is a global problem affecting approximately 69 million people annually [1–3]. Most clinical forms of TBI are closed-head diffuse brain injuries resulting from rapid rotational acceleration during falls, traffic collisions, or assault [4, 5] which may result in acute neurological deficits, life-long disability, or death [6–8]. As a consequence of the primary, mechanical insult to brain tissue, a number of deleterious biochemical cascades, collectively termed the secondary injury, diminish tissue health and can exacerbate pathology over time [9–13]. Brain injury due to rotational acceleration is dependent on brain mass and anatomy, and is therefore unique to humans and other large animals [9, 14]. Our group utilizes an advanced porcine model of closed-head rotational acceleration to model diffuse TBI and study neuropathology, glial reactivity, and recovery [15–25]. We have previously identified that neuronal cell bodies are vulnerable during trauma and can become transiently permeabilized [15, 19]. Neuronal membrane permeabilization could be an important initiator of secondary injury cascades because it is associated with released damage associate molecular patterns (DAMPs), dysregulated ionic gradients, and leaked excitotoxic molecules [9, 26–28]. Neuronal mechanoporation occurs in closed-head diffuse brain injury and can occur independently of blood–brain barrier damage [14, 22]. Previous work characterizing this model has demonstrated that different regions of the brain are heterogeneously affected by this type of neuropathology, with groups of permeabilized neurons exhibiting a “skip phenomenon” throughout the tissue, in which regions of localized pathology are spanned by other regions of seemingly unaffected tissue [15, 22]. However, the factors that influence this distribution of acute biophysical neuropathology resulting from head rotational TBI remain unknown. We postulated that structural differences—transitions in material properties—throughout the brain tissue may locally concentrate stresses and thus increase the likelihood for neuronal pathology. Specifically, we hypothesized that proximity to vasculature, which exhibits different mechanical properties from the surrounding brain parenchyma [29, 30], would locally increase the likelihood of neuronal permeability. We hypothesized that “perivascular regions” close to vessels might exhibit a higher burden of permeabilized neurons due to local mechanical property transitions relative to regions more distal to vasculature.

To test this hypothesis, we histologically examined the brains of swine following a sham, single, or repetitive TBI separated by 15 min, 3 days, or 7 days to ascertain the number and distribution of permeabilized neurons in relation to mid-size blood vessels (ranging in size from approximately 9–650 μm in diameter). Initial examinations focused on all anatomical structures in coronal brain sections (e.g., cerebral cortex, hippocampus, thalamus, etc.) to quantify the burden of permeabilized neurons across region and injury conditions. Thereafter, we sought to determine whether there was a *preferential* distribution of neuronal permeability in perivascular regions. We employed an unbiased sampling methodology that registered the position of all blood vessels as well as all permeabilized neurons in coronal sections to determine if neuronal permeability density increased based on proximity to vasculature. This more detailed methodology focused on midbrain regions because subcortical oculomotor nuclei within the midbrain—including the substantia nigra pars reticulata, caudate, and superior colliculus—are preferentially vulnerable to neuronal plasma membrane permeability following TBI [19]. These analyses were performed following both single and repetitive head rotational acceleration induced TBIs, as we and others have shown that repetitive rotational injuries exacerbate the extent of neuropathological burden, including the incidence of neuronal permeability using this model [15, 19, 22, 31, 32]. This study aims to increase our understanding of the potential relationship between heterogenous anatomical features and neuronal vulnerability to biophysical damage following high strain rate diffuse tissue deformations characteristic of TBI.

## Methods

### Animal handling and anesthesia

All animal procedures within this project were approved by the University of Pennsylvania’s Institutional Care and Use Committee, followed the ARRIVE guidelines, and were completed within an Association for Assessment and Accreditation of Laboratory Animal Care (AAALAC) accredited facility [33]. Female Yorkshire pigs (20–30 kg) were used within this study. All animals were purchased from Animal Biotech Industries and prior to experimentation were quarantined until veterinary staff could confirm the pigs were free of infection. Animals were fasted 12 h prior to TBI procedure but water remained ad libitum. Within this study, we utilized archival tissue from two separate porcine cohorts that had each been generated for separate TBI studies [15, 19, 22]. First, a group of 10 animals (n = 4 sham, n = 3 single TBI in the coronal plane, and n = 3 single TBI in the sagittal plane) was utilized for initial descriptions of the pathological phenomenon. Thereafter, a second group of 21 animals (n = 4 sham, n = 6 single TBI, n = 3 repetitive TBIs separated by 15 min, n = 4 repetitive TBIs separated by 3 days, and n = 4 repetitive TBIs separated by 7 days, all in the sagittal plane) was utilized to employ more detailed and systematic analyses [19]. Prior to surgical procedure, animals were induced with an intramuscular injection of ketamine (12–26 mg/kg) and midazolam (0.3–0.6 mg/kg). Animals were continuously monitored for normothermia and temperature was maintained through a forced warm air Bair Hugger system. Animals were intubated with endotracheal tube and anesthesia was maintained at 1–5% isoflurane driven with 2–3 L/min of 100% oxygen. Anesthesia was titrated throughout the experiment to maintain normothermia and vital signs (heart rate ranging from 100 to 130 beats/min, respirations from 9 to 12 breaths/min, and SpO_2_ between 97 and 100%). Animals were given 0.01 mg/kg glycopyrrolate subcutaneously and eye lubricant was applied.

### LY administration

Lucifer yellow (LY; Invitrogen, L453, Carlsbad, CA) was introduced into the lateral ventricles of all pigs on the terminal day to demarcate cells that were permeabilized during injury. LY is typically membrane impermeable and can only enter a cell’s intracellular space if membrane integrity is compromised. To administer intracerebroventricular LY to animal subjects, the surgical site was cleaned and shaved, and animals were positioned in a stereotactic head frame. Pigs were sterilely draped and aseptically cleaned with betadine, and the surgical team performed the procedure under sterile conditions. Briefly, a 4 cm incision was made on the midsagittal suture to expose the skull. Thereafter, two 5 mm craniectomies were drilled 1.0 mm posterior to the bregma and ± 6.0 mm lateral to the midsagittal suture. The site was cleaned with sterile saline, and a small incision was made in the dura. A sterile Hamilton syringe mounted in an arm of the stereotactic headframe was slowly lowered 18.0 mm from the surface into the brain to reach the lateral ventricles. Thereafter, an UltraMicroPump III (World Precision Instruments, Sarasota, FL) syringe pump pushed 500 µL of LY (0.4 mg/kg in sterile saline) into lateral ventricles over 10 min. The syringe was withdrawn at a rate of 2 mm/min. The procedure was repeated in the opposing lateral ventricle. The dye was allowed to diffuse throughout the brain’s parenchyma for 2 h from the start of the first LY injection. Craniectomy sites were packed with bone wax, the skin was sutured with 0-0 prolene sutures, and the area site was swabbed with betadine.

### Inducing closed-head diffuse TBI to pigs

Rotational acceleration closed-head diffuse TBI was completed on anesthetized animals by utilizing a HYGE pneumatic actuator. At the designated timepoint, the mouth of the anesthetized pigs was positioned around the HYGE’s padded bite plate. Oral soft tissue and teeth were protected with rubber padding and gauze while the pig’s snout was secured to the bite plate with adjustable snout cables. Immediately prior to injury, anesthesia was disconnected from the intubation tube and the animal’s head was rapidly rotated in the sagittal plane (in plane with the brainstem) from 80 to 130 radians per second or in the coronal plane from 245 to 300 radians per second [14]. The device induced rapid rotational acceleration of the subject’s head, scaled up to induce biomechanical parameters consistent with human TBIs [14, 18]. In general, rotation at these velocities in the coronal plane generates a less injurious TBI while rotation in the sagittal plane generates a more injurious TBI [14]. Angular displacement over time was recorded at a sampling rate of 10 kHz using hydrodynamic sensors (Applied Technology Associates, Albuquerque, NM) connected to a data acquisition system (DAQ; National Instruments, Austin, TX) that was controlled by LabVIEW (National Instruments). Sham animals received all other procedures absent head rotation.

### Recovery from anesthesia or euthanasia

Following TBI, pigs were either recovered or immediately euthanized. Animals that were allowed to recover were transported to clean housing units and extubated when prompted by chewing or swallowing. Animals were closely monitored for the duration of the recovery until completely ambulatory. At the terminal time point, animals were euthanized through transcardial perfusion with 0.9% heparinized saline followed by 10% neutral buffered formalin. Pigs experiencing a single sham, coronal, or sagittal TBI were sacrificed 15 min following the injury procedure. Subjects were decapitated and tissue was submerged overnight in formalin to ensure fixation. Brains were extracted and post-fixed in formalin for 5 days at 4°C. Brains were blocked coronally every 5 mm, frozen, stored at −80°C, and 20–25 µm sections were collected via cryostat.

### Immunohistochemistry

Immunohistochemistry was employed to determine LY positive (LY^+^) cellular phenotype and to verify that small voids within the brain parenchyma were indeed blood vessels. After washing and blocking in 4% normal horse serum with 0.3% Triton-X for 1 h at room temperature, LY^+^ tissue was incubated in blocking solution at 4˚C overnight with primary antibodies against alpha-smooth muscle actin (αSMA; 1:500, mouse, Abcam; #ab7817), a marker of pericytes; CD31 (1:100, rabbit, Novus Biologicals; #NB100-2284), a marker of endothelial cells; glial-fibrillary acidic protein (GFAP; 1:500, rabbit, Millipore; #AB5804), an intermediate filament enriched in astrocytes; and/or neurofilament, heavy subunit (NF-200; 1:300, mouse, Sigma-Aldrich; #N0142), a neuron-specific cytoskeletal constituent. Thereafter, tissue was incubated for 2 h with the appropriate secondary antibody (e.g., donkey anti-mouse 568 (1:1000, Invitrogen, A10037), donkey anti-rabbit 568 (1:500, Invitrogen, A10042), donkey anti-mouse 647 (1:500, Jackson ImmunoResearch, 715-605-151), and/or donkey anti-rabbit 647 (1:1000, Invitrogen, A31573)) at room temperature. Sections were preserved with Fluoromount-G (Southern Biotech) and coverslipped. A negative control (fluorescent secondary with no primary antibody) was employed to ensure correct interpretation of the fluorescent signal. Hematoxylin and Eosin (H&E) staining was completed on tissue to assess tissue structure, cellular morphology, and pathology after sham or rotational injury procedures.

### Imaging

Fluorescent images of non-tiled LY, αSMA, CD31, GFAP, and NF-200 stains were obtained using a Nikon A1Rsi Laser Scanning Confocal microscope with a 10x, 20x, or 60x objective (CFI Plan Apo Lambda 10x, N.A. = 0.45; 20x, N.A. = 0.75; 60x Oil, N.A. = 1.40). Tiled epifluorescence images of the porcine midbrain depicting LY cells were captured using a Nikon Eclipse Ti-S inverted epi-fluorescent scope outfitted with an Andor Zyla sCMOS 5.5 megapixel camera interfaced with Nikon Elements Basic Research software (4.10.01) with a 20x objective (Plan Apo Lambda 20x, N.A. = 0.75). The entire midbrain region of each subject was tiled with 15% individual tile overlap. Each brain hemisphere was captured in an independent image. H&E sections were imaged at 20x optical zoom using an Aperio CS2 digital slide scanner (Leica Biosystems Inc., Buffalo Grove, IL). All images acquired for comparative analysis were captured with identical acquisition settings.

### Neuropathological characterization

#### Presence of LY^+^ cells in regions of interest

**Fig. 1:**
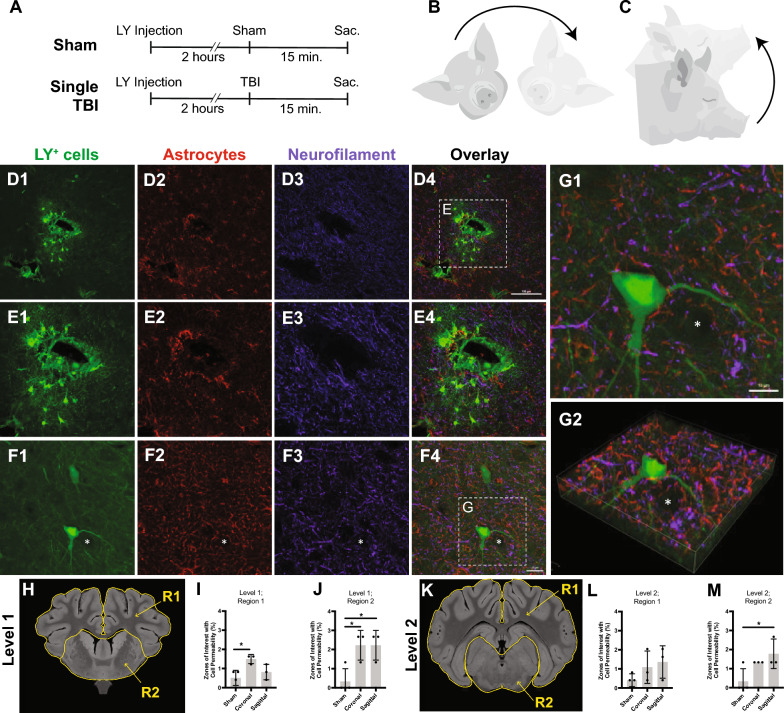
Neurons exhibiting alterations in plasma membrane permeability are present in the brain following closed-head rotational acceleration-deceleration induced TBI in pigs. **A** Animal subjects received either a sham injury or a single TBI prior to euthanasia. LY was administered intracerebroventricularly to pigs 2 h prior to the injury and sacrifice procedure. Anesthetized pigs receiving a TBI were subjected to rapid rotational acceleration in the **B** coronal or **C** sagittal plane. Confocal reconstructions from the ventral medial thalamus following a single diffuse TBI caused by head rotation in the sagittal (**D**–**E**) or coronal (**F**–**G**) plane. Lucifer yellow (LY) intracellular flooding and sequestration were used to denote cells with alterations in plasma membrane permeability. Sections show (1) permeabilized cells (LY^+^, green), (2) astrocytes (GFAP^+^, red), (3) neurofilaments (NF-200^+^, purple), and (4) overlay. (D) LY^+^ cells were observed in perivascular domains (scale bar = 100 μm), **E** with an absence of co-localization between LY, GFAP, and NF-200 staining as shown in high magnification reconstruction. **F** A permeabilized cell is observed wrapping around a smaller blood vessel (scale bar = 20 μm), **G** with 3D confocal reconstructions (scale bar = 10 μm). LY^+^ cells did not co-label with axonal or astrocytic markers. The percentage of LY^+^ zones was quantified for animals experiencing a sham, single coronal, or single sagittal injury (**H**–**M**) across a more rostral (**H**–**J**) or a more caudal level (**K**–**M**). The percentage of LY^+^ zones of interest was quantified across anatomical regions containing the cerebral cortex, hippocampus, and corpus callosum (referred to as Region 1, R1) or the thalamus and midbrain (referred to as Region 2, R2). Graphs plot data from each animal as a point, the group’s mean, and standard deviation (SD), where * denotes significant differences between groups where *p* < 0.05

The presence and density of LY^+^ cells were quantified in de-identified sections by experienced technicians who were both blinded to the tissue experimental group. For each animal, a total of 320 unique blood vessels were identified as zones of interest across multiple anterior–posterior regions grouped as follows: Region 1—corpus callosum (white matter), cerebral cortex (cortical gray matter, sub-cortical white matter, and hippocampus) and Region 2—deeper brain structures including midbrain, thalamus, and basal ganglia (gray and white matter; Fig. 1). Permeabilized neurons—expressing bright LY signal and containing visible neuronal characteristics—in the zones of interest were quantified based on the following inclusion criteria: (1) manually identified blood vessels were between 15 and 115 µm in diameter (a 95% CI designed to exclude capillaries and major blood vessels); and (2) the zones of interest were defined as the area within 125 µm of the vessel wall. A positive zone was defined as having at least one LY^+^ neural cell.

#### Distribution, density, and localization of LY^+^ cells

LY cells in tiled coronal slices of the porcine midbrain were manually identified by two trained technicians who were both blinded to the tissue experimental group. Specifically, all permeabilized neurons expressing LY signal were quantified. Technicians utilized MATLAB to log the coordinate location and pixel intensity of each permeabilized cell. The LY pixel intensity of each cell was normalized to the background intensity of the image that contained the cell. A single coronal slice from the left and right midbrain tissue of each animal was quantified. Between 83 and 799 neurons were counted for each animal.

### Automated blood vessel identification and characterization

Vessels in the porcine midbrain were identified and characterized utilizing a custom-built MATLAB code (Supp. Figure 1A-C). First, the image analysis code identified pixel regions of high contrast and binarized the image to distinguish vessels (void spaces) from tissue. Then, the code counted each blood vessel and measured vessel features including the area, minor axis diameter, major axis diameter, aspect ratio, and image coordinates. Each post-processed image was then manually inspected, and any inaccuracies were removed from blood vessel data. Between 678 and 1571 blood vessels ranging in size from 8.8 to 164 µm were counted for each animal.

### Relating blood vessels to LY neuron distribution and density

For each LY^+^ neuron, we performed a “nearest neighbor analysis” to calculate the distance to every individual blood vessel within the tissue, utilizing the distance transform minus the vessel’s minor axis radius. The minimum value, indicating the most proximal vessel, was reported.

$$D= \left[\sqrt{{\left({x}_{LY}-{x}_{vessel}\right)}^{2}{ + \left({y}_{LY}-{y}_{vessel}\right)}^{2}}\right]-{r}_{vessel}$$

where *D* is the calculated distance, *(x*_*LY*_*, y*_*LY*_*)* are the LY^+^ cell’s coordinates, *(x*_*vessel*_, *y*_*vessel*_*)* are the coordinates to the center of the lumen of the most proximal vessel, and *r*_*vessel*_ is the radius of the corresponding vessel’s minor axis. Likewise, for each blood vessel, we utilized the distance transform to calculate the distance to every individual LY neuron within the tissue. The minimum value, the most proximal LY neuron, was reported.

Next, for every blood vessel, we counted the number of LY^+^ cells in the perivascular zone, defined as a distance of less than 50 µm from the vessel wall. We normalized this LY^+^ cell count to blood vessel area because larger vessels will have larger perivascular areas compared to smaller vessels. We then counted the number of LY^+^ cells in the extraperivascular region, defined as a distance between 50 and 200 µm from the vessel wall. We again normalized this LY^+^ cell count to blood vessel area to accommodate for the relationship between vessel size and extraperivascular area size. We confined perivascular and extraperivascular regions to 200 µm from the vessel wall since most blood vessels are within 400 μm of the next nearest blood vessel, preventing neighboring blood vessels from confounding findings (Supp. Figure 2).

### Statistical analyses

Initial analyses were intended to ascertain the proportion of regions of interest containing neuronal permeability. Here, quantification of LY^+^ cells was completed in a cohort of 10 pigs. The percentage of LY^+^ zones of interest was plotted for each animal across two brain levels and two brain regions. Statistical differences between injury conditions were assessed with an ordinary one-way ANOVA with Tukey’s test for multiple comparisons.

Subsequent analyses were intended to determine if localization of LY^+^ cells was incidental or preferential. Here, we employed a different cohort of 21 pigs all injured in the sagittal plane. Statistical analyses investigating the number of LY^+^ cells or the number of blood vessels across injury conditions utilized an ordinary one-way ANOVA with Tukey’s post hoc analysis as a correction for multiple comparisons. Fluorescent intensity of LY^+^ cells was normalized to background fluorescent intensity and then each cell was plotted within each animal replicate. A nested one-way ANOVA with Tukey’s post hoc test was employed to detect significant differences between injury conditions where differences exist. Similarly, a nested one-way ANOVA with Tukey’s post hoc test was employed to detect significant differences in blood vessel diameters between injury conditions. Neuropathological density between perivascular and extraperivascular regions after repetitive TBIs separated by 3 days was assessed with a nested *t* test to determine significant differences between regions. Given that the vast majority of vessels are not close to any LY^+^ signal, a nested *t* test was also performed on neuropathological density between perivascular and extraperivascular regions after excluding vessels that had no LY^+^ cells in either the perivascular or extraperivascular spaces.

To determine if blood vessel size or if blood vessel orientation influenced the number of proximal LY^+^ cells, we binned all blood vessels for each animal replicate experiencing repetitive TBIs separated by 3 days according to size or according to aspect ratio. The number of LY^+^ cells within 50 µm of each vessel was reported. After selecting only the blood vessels within 200 µm of an LY^+^ cell, the median number of LY^+^ cells within each bin was pooled across the animal replicates. Not all animals had LY^+^ blood vessels of all sizes, therefore bins with n = 1 were excluded because variance could not be estimated and assumptions of ANOVA could not be met. Data were assessed using a one-way ANOVA with Tukey’s post hoc test to determine if vessels of a specific size or orientation were more likely to colocalize with LY^+^ pathology. For all tests, the type I error rate was set to 0.05 and all hypothesis tests were two-sided. All statistical analyses were carried out in Prism Version 9.3.1 (350).

## Results

### Closed-head diffuse TBI in pigs generates neuronal permeability after a single TBI

Acutely after a single closed-head diffuse TBI in either the coronal or the sagittal plane (Fig. 1A–C), LY^+^ cells—indicative of cells with permeabilized plasma membranes—were observed in close proximity with mid-sized blood vessels (Fig. 1D–G). In agreement with previous studies, the LY^+^ signal did not colocalize with the astrocytic marker, GFAP, or the axonal marker, neurofilament [15, 22]. Taken with their unique morphology, these data suggest that these LY^+^ cells were exclusively neuronal and primarily labeled the somatic cell structures. In addition to neuronal mechanoporation, rotational injury generated intraparenchymal bleeds and hyperpigmented neurons in the midbrain (Supp. Figure 3).

Our initial goal was to quantify the zones containing permeabilized (LY^+^) neurons across brain regions and injury conditions. Here, we employed a *biased* analysis where we manually identified blood vessels as our zones of interest and quantified the presence of LY^+^ cells within 125 µm, a relatively large area that collectively covers a significant area of the brain. We then calculated the percentage of zones of interest that contained at least one LY^+^ neuron across various brain regions following three injury conditions (sham loading compared to head rotation in the coronal or sagittal plane). We found the incidence of LY^+^ zones were increased across anterior cerebral cortex, corpus callosum, and hippocampal regions following head rotation in the coronal plane, and in the anterior thalamus and midbrain following head rotation in either the coronal or sagittal plane (Fig. 1). Within these injury conditions, LY^+^ zones were generally most prevalent in the thalamus and midbrain regions following sagittal plane rotation (Fig. 1H–M). Given our interest in determining patterns of LY pathology, we focused subsequent analyses on this thalamic/midbrain region following sagittal plane rotation, with its increased LY^+^ burden to allow easier detection of pathological distribution patterns.

### Diffuse TBI generates patches of neuronal permeability in the midbrain

**Fig. 2:**
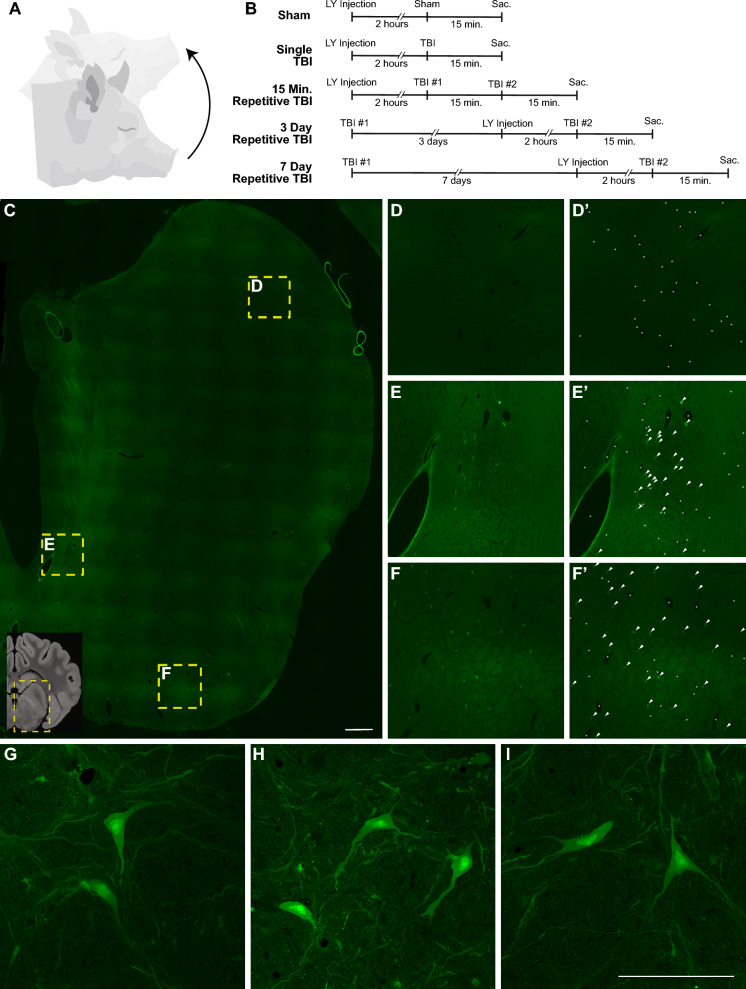
Diffuse TBI paradigm generates neuronal membrane permeability in the porcine midbrain. **A** Anesthetized pigs receiving a TBI were subjected to rapid rotational acceleration in the sagittal plane to generate diffuse damage. **B** Animal subjects received either a sham injury, a single TBI, repetitive TBIs separated by 15 min, repetitive TBIs separated by 3 days, or repetitive TBIs separated by 7 days prior to euthanasia. LY was administered intracerebroventricularly to pigs 2 h prior to the terminal injury and sacrifice procedure (Sac.). **C** LY highlights permeabilized neurons in the brain, allowing identification of regions **D**–**D**′ absent or **E**–**F**′ containing LY^+^ neurons. The dim grid pattern is a tiling artifact. **D**′–**F**′ Blood vessels (asterisks) are numerous in all images while LY^+^ neurons (arrowheads) exhibit a multifocal organization. (**E', F'**) LY^+^ neurons are easily identifiable in the brain tissue due to their bright fluorescence and morphological characteristics. Scale bars represent 500 µm (**C**) or 100 µm (**G**–**I**)

The previous observations did not distinguish between the possibility of neuronal pathology incidentally occurring near vessels versus neuronal pathology preferentially occurring near vessels. To investigate this, it was essential to compare the pathological burden in regions close to vessels and contrast them to regions further from vessels. As such, a separate cohort of pigs was utilized that was subjected to closed-head diffuse TBI through rapid rotational acceleration in the sagittal plane (Fig. 2A). Here, pigs experienced sham, single, or repetitive TBIs. Repetitive TBIs were separated by 15 min, 3 days, or 7 days and all animals were sacrificed within 15 min of final TBI procedure (Fig. 2B). As with our initial study, intracerebroventricularly administered LY fluorescently labeled permeabilized neurons in the midbrain of pigs. Similar to previous reports [15, 19], we observed a so-called “skip phenomenon” of pathology throughout our region of interest (Fig. 2C), where some regions exhibited no evidence of this pathology (Fig. 2D–D′) and other regions showed high numbers of LY^+^ cells (Fig. 2E–F′). LY neurons were identified by their characteristically brighter fluorescence relative to the tissue background and their morphology, many times exhibiting LY^+^ neurites in addition to LY^+^ somata (Fig. 2G–I).

**Fig. 3:**
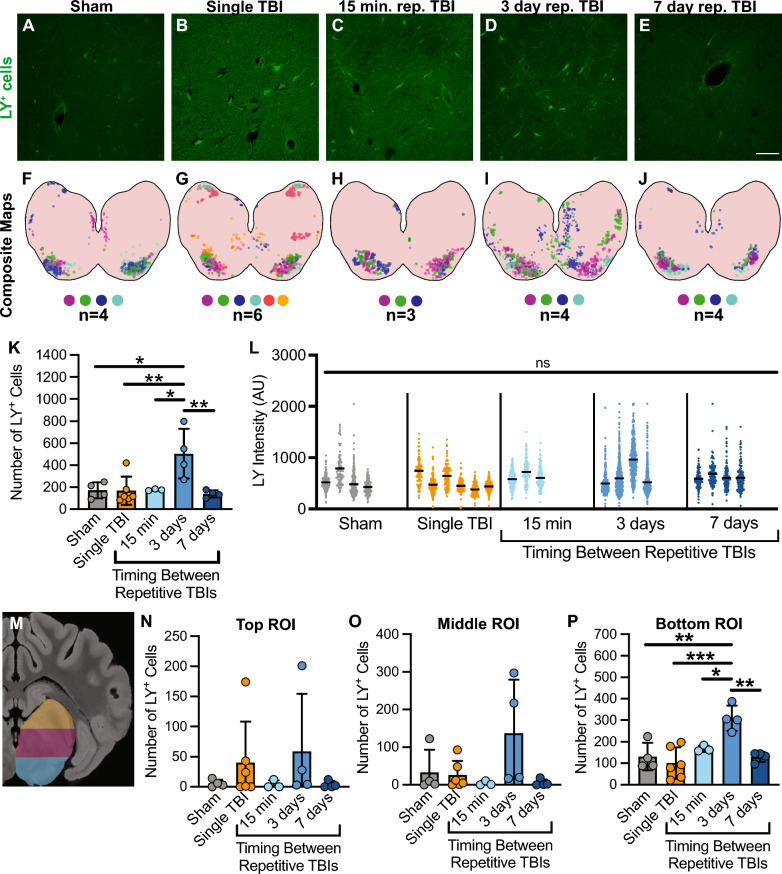
The total number of permeabilized neurons in the porcine midbrain is augmented in the 3-day repetitive TBI condition. **A**–**E** Representative images of LY^+^ neurons (green) visible in the midbrain of a **A** sham, **B** single TBI, **C** 15-min repetitive TBIs, **D** 3-day repetitive TBIs, and **E** 7-day repetitive TBIs conditions. **F**–**J** Composite maps indicate the distribution of individual LY^+^ neurons in animal replicates for each injury condition. Within each composite map, colored dots represent the coordinates for LY^+^ neurons from each animal subject within that condition. **K** The total number of LY^+^ cells counted across the midbrain for each animal in each condition was significantly upregulated in the 3-day repetitive TBI condition (mean ± SD). **L** The fluorescent intensity of each individual LY^+^ cell normalized to background intensity was not significantly affected by injury condition. Columns represent individual animal subjects within a condition and colors represent injury conditions. Horizontal black lines within each column represent the mean LY^+^ cell intensity for each animal. **M** The midbrain was divided into top (orange), middle (purple), and bottom (blue) thirds. The number of LY^+^ neurons in the **N** top and **O** middle regions of interest exhibited no significant effects across injury conditions. **P** The bottom region of interest exhibited a significant increase in LY^+^ neurons in animals experiencing repetitive TBIs separated by 3-day relative to all other injury conditions. “ns” denotes no significant differences across groups, *denotes significant differences between groups where *p* < 0.05, and **denotes significant differences between groups where *p* < 0.01. Scale bar represents 100 µm

All animals, including sham-injured animals, exhibited LY^+^ neurons in the midbrain (Fig. 3A–E). We qualitatively observed that LY^+^ neurons varied in number, location, and fluorescent intensity. To better understand the distribution of LY^+^ cells within the midbrain of animals across injury conditions, we counted every LY^+^ neuron within the midbrain from all animal subjects. Coordinates from each LY^+^ neuron were projected and overlayed across replicates within an injury condition to generate composite maps (Fig. 3F–J). Animal replicates were projected onto composite maps with different colors to illustrate variability and reproducibility between subjects. It is important to note the injury conditions contained between three and six animal replicates so the pattern, not the quantity, of colored points should be considered. These composite maps assisted in identifying regions of higher neuropathology across the relatively large area of the porcine midbrain.

We observed that all animals exhibited LY^+^ cell permeability in the ventral portion of the midbrain. In some injury conditions, animals also exhibited LY^+^ permeability in more medial and dorsal areas of the midbrain. Quantification of the total number of LY^+^ cells within each midbrain demonstrated that pigs experiencing repetitive TBIs separated by 3 days bore the largest amount of neuropathological burden (Fig. 3K). No significant differences were detected between any other injury conditions.

We previously observed that the fluorescent intensity of the LY^+^ cells varied within and between subjects. We postulated that more severe or repetitive injuries would generate more fluorescent LY^+^ neurons by mechanically permeabilizing vulnerable neurons to a greater degree, thus allowing more fluorescent dye to leak into and label the cells. Therefore, we measured the fluorescent intensity of each individual LY^+^ neuron from each midbrain tissue section. Thereafter, we subtracted out the background fluorescent intensity by animal, which may vary depending on perfusion parameters. While there was large variability in LY^+^ cell fluorescent intensity within animal subjects, we did not detect any significant differences in LY^+^ cell intensity across injury conditions (Fig. 3L).

To better understand the distribution of LY^+^ neuropathology, we trifurcated the porcine midbrain into three regions of interest. The top region of interest represents the most dorsal third of the midbrain (orange; Fig. 3M), the middle region of interest characterized the middle third of the midbrain (purple; Fig. 3M), and the bottom region of interest contains the most ventral third of the midbrain (blue; Fig. 3M). LY^+^ neuron counts were not significantly affected across injury condition in the top and middle regions of interest (Fig. 3N, O). We found that only the bottom region of interest in the 3-day repetitive condition exhibited a significant increase in LY^+^ cell permeability relative to other injury conditions (Fig. 3P).

### Large blood vessel numbers and density decrease following repetitive TBIs in pigs

We sought to concatenate information on vasculature throughout the porcine midbrain to test our hypothesis relating the distribution of neuropathology to vasculature. As previously noted, because the LY dye diffuses throughout the entire brain prior to injury, it causes background autofluorescence of the tissue. The hollow lumen of vasculature remains unstained during this labeling process allowing for easy identification of individual vessels (Supp. Figure 4A). To validate that these LY^−^ voids in the brain tissue were indeed vessels, we stained for the endothelial cell marker CD31 and the pericyte marker αSMA (Supp. Figure 4A-B’). We observed that the black voids within the LY-stained tissue exhibited CD31^+^ and αSMA^+^ perimeters (Supp. Figure 4A-B’), suggesting that endothelial cells and pericytes lined these vessels. LY^−^ voids were CD31^+^ and αSMA^+^ across all injury conditions (Supp. Figure 4C-G’). 417 LY^−^ voids were individually assessed to determine generalizability of blood vessel identification. Over 99% of LY^−^ voids colocalized with CD31 and/or αSMA, confirming that LY^−^ voids identified with these metrics were blood vessels.

**Fig. 4:**
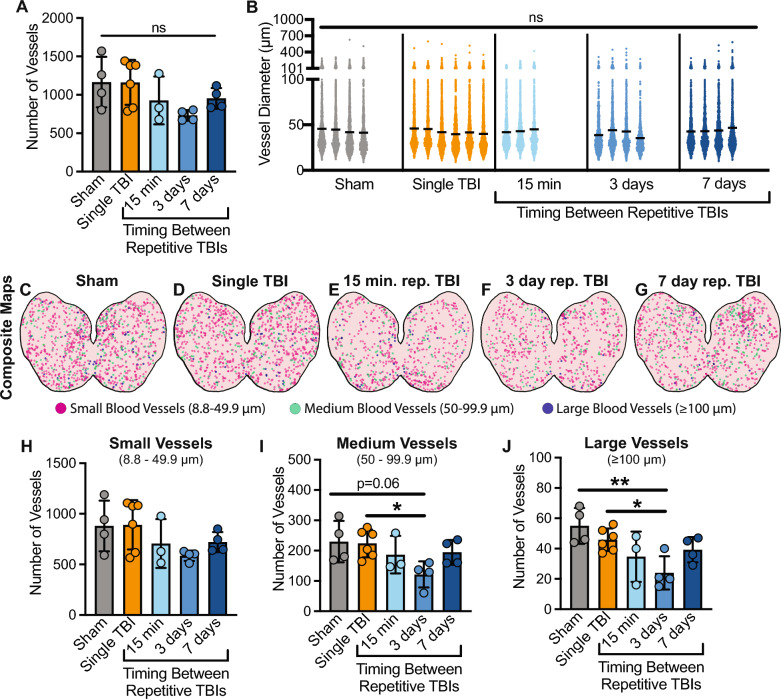
The number of large blood vessels decreased after repetitive injuries separated by 3 days. **A** The total number of blood vessels across the midbrain for each animal was not significantly affected across injury conditions although there was a trend toward decreased numbers of blood vessels within the 3-day repetitive TBI condition (mean ± SD). **B** The mean vessel diameter of each individual blood vessel was not significantly affected by injury condition. Each column represents an animal subject and colors represent injury conditions. Horizontal black lines within each column represent the mean blood vessel diameter for each animal. **C**–**G** Composite maps of small, medium, and large blood vessels from one representative animal in each injury condition. The total number of **H** small, **I** medium, and **J** large blood vessels was quantified across all injury conditions. “ns” denotes no significant differences across groups, *denotes significant differences between groups where *p* < 0.05, and **denotes significant differences between groups where p < 0.01

Utilizing the LY^−^ staining, we developed a custom image analysis strategy to identify vasculature throughout the porcine midbrain (Supp. Figure 1A-C). Manual validation of this semi-automated strategy identified between 678 and 1571 blood vessels within each tissue section. The identified vessels ranged in size from 8.8 to 626.2 µm with more than 99% of vessels greater than 15 µm in diameter. To understand the distribution of vasculature throughout the porcine midbrain across injury conditions, again we generated composite maps (Supp. Figure 1D-H). Vessel coordinates from animals within an injury condition were overlayed to determine vascular distribution across the midbrain. Importantly, the number of animals in each injury condition ranged from 3 to 6 so the point distribution rather than the point density should be considered. We observed that vasculature was relatively uniform throughout the midbrain. Quantification of the total vasculature within each midbrain generated an interesting trend. We found that there was a non-significant decrease in the total number of vessels in the midbrain of pigs experiencing repetitive TBIs separated by 3 days (Fig. 4A). This trend of decreasing vessel numbers was not significant between sham and 3-day repetitive TBIs (*p* = 0.11) or between single injury and 3-day repetitive TBIs (*p* = 0.16).

To further investigate if the vasculature was changing, we investigated if blood vessel diameters were different across injury conditions. While there was notable variability in blood vessel sizes within each subject’s midbrain, we did not observe significant differences between injury conditions when all vessel sizes were considered together (Fig. 4B). Plotting the vascular distribution from a representative animal in each injury condition indicated there may be a change in vessel density across injury condition (Fig. 4C–G). Therefore, vessels sizes were categorized as small vessels (< 50 µm), medium vessels (50–99.9 µm), or large vessels (≥ 100 µm) based on their diameter. We observed no significant changes in the number of small vessels across injury conditions (Fig. 4H). We did observe a significant decrease in the number of medium-sized vessels between a single TBI and repetitive injuries separated by 3 days and a non-significant decrease in the number of medium vessels between sham and repetitive injuries separated by 3 days (*p* = 0.06; Fig. 4I). Furthermore, we observed a significant difference in the number of large blood vessels in the midbrain of pigs experiencing repetitive injuries separated by 3 days relative to sham and single injury conditions (Fig. 4J). In line with these findings, we found that large vessel density in the midbrain significantly decreased in animals experiencing repetitive injuries separated by 3 days relative to sham animals (Supp. Figure 5).

### Neuronal mechanoporation is preferentially colocalized with midbrain vasculature in regions of vulnerability

**Fig. 5:**
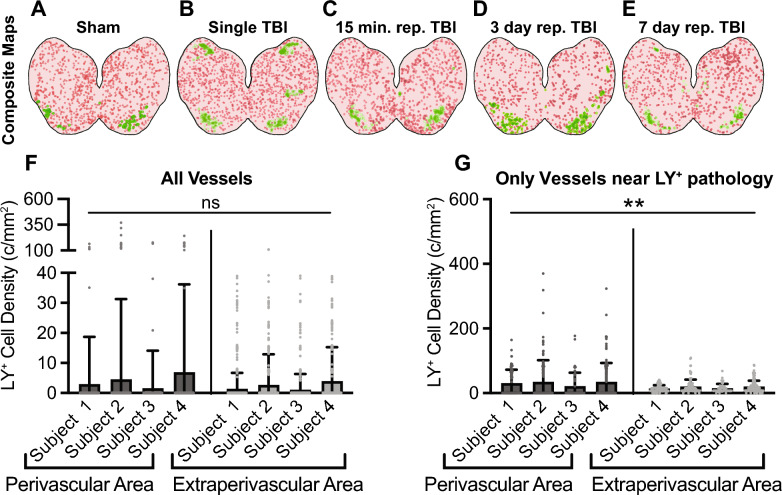
LY^+^ neurons are preferentially associated with blood vessels in regions with neuropathology. **A**–**E** Composite maps were generated from one representative animal within each injury condition. Coordinates for blood vessels (red points) and coordinates for LY^+^ neurons (green points) were overlayed to illustrate midbrain distribution of blood vessels and LY^+^ cells relative to each other across **A** sham, **B** single TBI, **C** 15-min repetitive TBIs, **D** 3-day repetitive TBIs, and **E** 7-day repetitive TBIs conditions. Within the 3-day repetitive TBI condition, the area around each blood vessel was divided into two concentric rings with the more proximal ring (within 50 µm of the vessel boundary) defined as the perivascular area and the more distal ring (between 50 and 200 µm from the vessel boundary) defined as the extraperivascular area. The LY^+^ density in each area was quantified for each blood vessel. **F** No significant differences were detected when comparing the perivascular area to the extraperivascular area when considering all vessels in the midbrain. **G** When only considering LY^+^ vessels, there was a significant enrichment in LY^+^ cell density in perivascular relative to the extraperivascular areas. “ns” denotes no significant differences across groups and **denotes significant differences between groups where *p* < 0.01

Given that we compiled coordinates for each LY^+^ neuron and each blood vessel within each subject’s midbrain, we next considered our main hypothesis. We first generated composite maps to visualize if LY^+^ neurons co-localized with blood vessels (Fig. 5A–E). These composite maps only plot vessels and LY^+^ neurons from one representative animal within each injury condition where red points represent vessels and green points represent LY^+^ cells. We observed that while the distribution of midbrain vasculature was relatively homogenous, the distribution of LY^+^ neurons was heterogenous and appeared to be clustered in discrete anatomical regions at the base of the midbrain and, occasionally, in the dorsal region. We next focused on the distributions within the 3-day repetitive TBI condition—a condition exhibiting significantly different levels of LY^+^ neurons under these experimental conditions—by calculating the distance from each LY^+^ cell to the nearest identified blood vessel. We found that the median distance from each LY^+^ cell to the nearest vessel was between 241 and 424 µm (Supp. Figure 2B); note that the size range of vessels detected in this study excludes most, if not all, capillaries. Thereafter, we calculated the distance from each blood vessel to the nearest LY^+^ cell. We found that the median distance from each vessel to the nearest LY^+^ cell was between 964 and 2,302 µm (Supp. Figure 2C). Taken together, these data suggests that LY^+^ cells are always somewhat close to a vessel, but vessels do not necessarily co-localize with LY^+^ neurons.

To determine if perivascular regions directly proximal to vessels are more likely to exhibit LY^+^ neuropathology, we determined the LY^+^ cell density in regions close to or further away from vessels. We defined the perivascular area as the concentric area that extended 50 µm beyond the boundary of a vessel wall. We defined the extraperivascular area as the concentric ring that extended from 50 to 200 µm beyond the boundary of a vessel wall. We chose these regions because vessels are typically within 400 µm of another vessel (Supp. Figure 2A). Therefore, to avoid analyzing regions that were influenced by multiple vessels, we defined perivascular and extraperivascular regions within half the distance to most adjacent vessels. Within animals experiencing repetitive TBIs separated by 3 days, we found that there was no significant difference in LY^+^ cell density in the perivascular area relative to the extraperivascular area when considering all vessels, indicating that perivascular location was not the primary driver of LY^+^ pathology location (Fig. 5F). However, the majority of vessels in the midbrain do not associate with any LY^+^ neurons. Given that neuronal mechanoporation demonstrated a strong regional preference—with most pathology associated with the ventral midbrain—we considered if there was a secondary perivascular pathology effect within regions that were prone to neuronal mechanoporation. Therefore, we analyzed the LY^+^ density in perivascular and extraperivascular areas only for vessels that were close to at least one LY^+^ neuron. All vessels that had at least one LY^+^ neuron in either the perivascular or extraperivascular spaces were included. Using this more focused assessment, we found that perivascular regions had a significant increase in LY^+^ cell density relative to extraperivascular areas (Fig. 5G). Together, these data indicate that while LY^+^ pathology is affected by vessel proximity, it is not the primary determinate of its spatial distribution. These findings support a model in which perivascular proximity enhances local neuropathology in already vulnerable regions rather than initiating it.

### Neither blood vessel size nor orientation drives LY^+^ neuropathology

To further investigate our main hypothesis, we considered if other characteristics of the vasculature could be more likely to increase localized neuropathology. Indeed, Monson et al. (2019) speculated that vessels oriented such that they would experience axial strain during TBI are most likely to exhibit vessel dysregulation at subfailure thresholds [30]. We also speculated that larger vessels might exhibit differing mechanical properties relative to smaller vessels. Thus, we investigated if vessel orientation or size were associated with localized neuropathology. First, we considered neuropathology across vessel sizes because vessels exhibited a wide range of diameters (Supp. Figure 6). We postulated that if the mechanical properties of vessels affect LY^+^ neuropathology, larger vessels, which have thicker vessel walls, could exaggerate this effect. We completed this analysis using histology data from each animal experiencing repetitive TBIs separated by 3 days. Because our previous analysis indicated that perivascular effects were subtle and could only be detected within vessels that had some LY^+^ neurons in proximity, we excluded all vessels that had no LY^+^ cells within 50 µm of the vessel wall. We found no significant effect of blood vessel size on the number of LY^+^ cells within 50 µm of each vessel (Supp. Figure 6B).

Finally, we postulated that the orientation of vasculature within the midbrain could affect neuropathology. Because these analyses were performed on porcine tissue following closed-head diffuse TBI generated by rotating in the sagittal plane (but the tissue was subsequently sectioned in the coronal plane), we suspected that vessels oriented more parallel with the field of view (i.e., locally running within the coronal plane) could generate different tissue level mechanics than vessels oriented perpendicular to the field of view (i.e., locally running orthogonal to the coronal plane); these vessel orientations would cause principal shear strains to be oriented longitudinally versus axially, respectively, with respect to the long axis of the blood vessel. To determine this, we considered the aspect ratio of each vessel, which was defined as the long axis normalized to the short axis of the vessel. A higher aspect ratio indicates that a vessel is locally oriented parallel (i.e., within) the coronal plane. An aspect ratio of 1 represents a vessel that is perfectly circular, meaning that the vessel is locally running orthogonal to the coronal plane. The highest aspect ratio measured was 16 (i.e., a vessel that is 16 times longer than it is wide within the tissue section), and values ranging between 1 and 16 indicate various degrees of obliqueness in the local vessel trajectory with respect to the coronal plane. Applying this methodology allowed consideration of LY neuropathology across vessel aspect ratios ranging from 1 to 16. As above, we calculated the median number of LY^+^ cells across vessel aspect ratios for animals experiencing repetitive TBIs separated by 3 days, excluding vessels that had no LY^+^ cells. Like blood vessel size, we found no significant effect of blood vessel aspect ratio on the number of LY^+^ cells within 50 µm of each vessel (Supp. Figure 6C).

## Discussion

Previous investigations by our group have reported changes in neuronal membrane permeability caused by a diffuse closed-head rotational acceleration TBI in pigs [15, 19, 22]. These studies used intracellular flooding with LY—a normally cell-impermeant fluorescent marker—to denote neurons presumed to have experienced mechanoporation by the traumatic shear stresses associated with closed-head rotational TBI. We found that the distribution of LY^+^ neurons acutely after TBI followed a so-called “skip phenomenon” throughout the brain, defined as a heterogenous distribution whereby clear patches of LY^+^ neurons were identified that were spanned by regions exhibiting an absence of LY^+^ neurons. In addition, the size and frequency of these patches of LY^+^ neurons were generally increased after repetitive injuries [15, 19, 22]. The primary goal of the current study was to revisit archival tissue from a subset of these previous studies to ascertain whether distinct microanatomical features, such as vascular distribution, could explain this heterogenous distribution of LY^+^ neurons.

Accordingly, we histologically examined the brains of pigs following a sham, single, or repetitive TBI separated by 15 min, 3 days, or 7 days to ascertain the density and distribution of permeabilized neurons in relation to blood vessels ranging in size from approximately 8-650 μm. Initial examinations focused on a range of anatomical structures (e.g., cerebral cortex, hippocampus, thalamus, etc.) following a single head rotational acceleration in the coronal or sagittal plane, and the analysis involved the random selection of zones of interest to quantify the presence of permeabilized neurons. This initial methodology revealed that there were statistically significant increases in the percentage of zones containing LY^+^ neurons following head rotation in either the coronal or sagittal plane; however, these changes were most pronounced in the thalamus-midbrain following head rotation in the sagittal plane.

While this initial methodology was suitable to describe the *incidence* of neuronal permeability in perivascular regions, it was not able to ascertain whether there was a *preferential* distribution of neuronal permeability in perivascular regions. Therefore, we next employed an unbiased sampling methodology that registered the position of all blood vessels as well as all permeabilized neurons to statistically determine if the probability of regions exhibiting neuronal permeability increased based on proximity to vasculature. This more detailed methodology only focused on the thalamus-midbrain region following head rotation in the sagittal plane; however, both single and repetitive injuries were examined. This analysis revealed that LY^+^ neuropathology—indicative of neuronal plasmalemmal damage—was elevated in the ventral region of the midbrain after repetitive TBIs separated by 3 days. Focusing on this experimental group, we statistically evaluated the putative relationships between the distribution of permeabilized neurons and blood vessels, which revealed a preferential distribution of LY^+^ neurons in proximity to blood vessels but only when vessels that were not associated with any LY^+^ neurons were excluded. These analyses showed that neuronal plasmalemmal permeability was not primarily driven by proximity to vasculature. However, within regions that had LY^+^ pathology, the density of LY^+^ neurons was increased in perivascular spaces relative to more distal spaces. Collectively, this suggests that perivascular spatial associations preferentially increase LY^+^ pathology density, but perivascular localization is not the primary driver of LY^+^ distribution.

These findings suggesting an increase in the quantity of LY^+^ neurons in the midbrain of pigs experiencing repetitive TBIs separate by 3 days are in line with previous literature suggesting that there is window of enhanced vulnerability after a TBI when subsequent trauma can exacerbate pathology [34–39]. Indeed, several other studies across a range of preclinical models have observed that TBIs separated by 3 days can amplify the extent of neuropathology [19, 40, 41]. For example, in a weight drop TBI model in a rat, researchers found that repetitive injuries separated by 3 days were associated with larger lesion volumes, more cortical damage, increased extravascular iron, increased glial activation, and a reduction in behaviors including spatial learning capacity relative to injuries separated by larger times [40]. In the same porcine cohort of diffuse TBI employed in the current analyses, previous analyses revealed increased neuropathology following repetitive injuries compared to sham injuries predominately in more rostral or in more caudal regions of the brain [19]. While Keating et al. investigated discrete nuclei [19], we strove to investigate a broader region and thus, complete analysis via unbiased region sampling. However, our unbiased sampling approach led us to the ventral midbrain in which Keating et al*.* also focused one analysis due to the presence of the substantia nigra pars reticulata and its importance in oculomotor function. When restricting analyses to discrete anatomical nuclei within this midbrain region, the group detected significant increases in LY^+^ neurons in repetitive injuries separated by 15 min or 3 days, suggesting nuclei-level comparisons may be more sensitive to detecting LY differences than more gross examinations initially employed in the current study. Thus, previous analyses find further validation through unbiased reproduction and additional context given new consideration of the role of neurovasculature. Furthermore, these findings, including the presence of LY^+^ neurons in the ventral midbrain of sham animals, suggest some variable properties of discrete brain nuclei (whether physiological, anatomical, cellular, or molecular) may affect the vulnerability of neurons to permeabilization during diffuse closed-head brain injury. We interpret the presence of LY^+^ neurons in the ventral midbrain of sham animals as reflecting an intrinsic vulnerability of this region to membrane perturbation during this procedure, which is selectively amplified only when repetitive injuries occur with a 3-day separation—suggesting that this interval uniquely coincides with unresolved biochemical or metabolic susceptibility in ventral midbrain neurons that is not present at shorter (15-min) or longer (7-day) inter-injury intervals.

Thus, the question remains: what is the primary factor that influences neuronal mechanoporation during rotational acceleration TBI? While perivascular proximity appears to play a secondary role, our findings suggest other determinants are more critical. This effect is time-dependent, as repetitive injuries separated by 3 days substantially increase the presence of permeabilized neurons relative to single injuries, consistent with a known window of vulnerability. The presence of LY^+^ neurons in sham animals indicates that some intrinsic property of certain regions may predispose neurons to mechanoporation. Although others have reported that LY can be internalized by neurons through endocytic mechanisms [42, 43], these observations occurred under atypical conditions (e.g., calcium-free environments) that do not reflect the injured brain. In contrast, we interpret LY labeling in the current study primarily as an indicator of physical membrane compromise, rather than via an active uptake mechanism. Future work should identify the intrinsic and extrinsic factors—such as neuronal subtype, extracellular matrix organization, lipid peroxidation, edema, and glial changes—that govern susceptibility to mechanoporation [40, 44, 45].

Intracerebroventricular administration of LY also necessitates consideration of physiological cerebrospinal fluid (CSF)-interstitial fluid (ISF) exchange pathways, including glymphatic transport. Following ventricular infusion, solutes in the CSF flow through the cisterna magna into the subarachnoid space and then enter the brain via periarterial spaces surrounding penetrating arteries, mix with ISF throughout the parenchyma, and subsequently drain along intramural perivenous pathways. Thus, the presence of diffuse background LY signal likely reflects normal CSF-ISF exchange and regional differences in solute exposure or clearance rather than injury-induced blood–brain barrier disruption.

In addition to observations related to the distribution of LY^+^ neurons, we also report changes to the porcine vasculature in the midbrain after TBI. We unexpectedly observed a reduction in the number of large (defined as ≥ 100 µm) and medium (defined as 50-99.9 µm) blood vessels in the midbrain of animals that experienced repetitive TBIs separated by 3 days, but not other injury groups. These decreases in vessel numbers did not appear to be confined to regions with LY^+^ neurons, rather, vessel loss was apparent across the whole midbrain. These findings are in line with previous studies in rats and humans which have reported changes to the vascular network after TBI [46–50]. For example, vascular corrosion casts were created from the brains of patients following fatal TBI, and analyses of images from scanning electron micrographs and immunohistochemistry revealed extensive damage to arterioles and capillaries in middle and deep cortical zones [50]. In a controlled cortical impact (CCI) model, researchers reported vascular changes at the injury site, in both the perilesional area and the contralateral hemisphere at acute timepoints [46]. Indeed, vessel junctions, vessel lengths, and biological complexity were all decreased following TBI [46]. Separately, two weeks after a fluid percussion injury (FPI), microvascular density in the cortex was decreased, although not significantly, relative to sham animals [47]. Moreover, repetitive injuries may exaggerate these neurovascular pathologies. In a rat model of mild repetitive blast TBI, researchers observed changes to the cerebral vasculature 6 weeks post injury including swelling of perivascular astrocytes which may affect circulation and tissue perfusion [48]. Additionally, Gama Sosa et al. reported persistent, widespread vascular pathology including thinning of the vascular density and vascular organization 10 months after repetitive blast TBIs [48]. Finally, a study of diffuse TBI in non-human primates found slowed cerebral blood flow and smaller vessels following rotational TBI [51].

To our surprise, the number of larger, not smaller, vessels was decreased in the midbrain after repetitive TBIs separated by 3 days. We speculate that there could be four factors causing this observation. First, larger vessels could be more susceptible to injury biomechanics because larger vessels may fail at lower gradients of stress/strains relative to smaller vessels. Second, vessels could change their mechanical properties as a consequence of injury and subsequently, there could be a differential response to the fluid pressure of the transcardial perfusion procedure. Third, all the vessels in the midbrain could undergo contraction, thus reducing the numbers of large vessels without inflating smaller vessel populations. Fourth, microthrombi within the largest vessels could be coating vessel walls, decreasing the apparent vessel lumen size [52].

While we cannot definitively exclude any of these possibilities, we suspect that latter option of microthrombi partially occluding brain vasculature would be exceptionally obvious to detect because red blood cells exhibit autofluorescence at these wavelengths. In contrast, we suspect the three former options could generate the data patterns reported here. In considering the first option, when larger vessels fail, they can result in parenchymal hemorrhage, which we did not observe, or they could initiate vascular involution. Vascular involution, defined as an entire vessel network closing off and regressing, proceeds by vessel stenosis, apoptosis, and basement membrane degradation, respectively. Stenosis can be induced via vessel stretching or tumor necrosis factor alpha (TNFα) signaling—triggers which are present during and after TBI—and results in narrowing of vessel diameters [53]. Thereafter, apoptosis and degradation of the basement membrane of midbrain vessels would decrease the vessel populations. When considering the second option, vessels can change their mechanical properties in response to subfailure threshold strains. Subtle shifts in vascular size distributions could be an artifact of graduations in responses to perfusion pressures associated with transcardial perfusion. Live animal neuroimaging, for instance using vessel contrast dyes, may be necessary to validate our findings and rule out that vascular changes are an artifact of the peri-mortem perfusion procedure. Likewise, when considering the third aforementioned possibility, abrogated vessel contractility and increased coagulation has been well documented in TBI across injury type and preclinical animal models [54, 55]. We speculate that our findings could be explained if all midbrain vessels were undergoing contraction after repetitive TBIs separated by 3 days. Indeed, this would decrease the number of observable larger or medium vessels in the tissue and the smallest capillaries (< 8.8 µm), which cannot be detected with these metrics, would not show significant changes. We suspect that changing vessel mechanics, vascular involution, and abrogated vessel contractility, or a combination of these, may be drivers of these findings because they are associated with time-dependent changes.

Cerebral vascular contraction and tissue hypoperfusion due to trauma could be the reaction to biochemical secondary injury signaling in the brain parenchyma and/or could be the result of direct damage to the vessels during injury. There are many candidate pathways within the secondary injury cascade that could affect vessel contractility including release of reactive oxygen species [56], endothelins [57, 58], and arachidonic acid derivatives [59]. Furthermore, damage to the vessels themselves during the mechanical insult induces a complex cascade of biochemical signals changing the endothelial lining from an anti-inflammatory and anti-coagulatory surface to a pro-inflammatory and pro-coagulatory surface (for thorough review, please see [60]). Vessel rupture initiates coagulation, complement signaling [61], and cytokine signaling resulting in vascular inflammation, perturbations to vessel contractility, increases the endothelial cell–cell junction size, initiation of microthrombi formation, and altered tissue perfusion efficiency [60]. Indeed, even following subfailure deformations, where vessels undergo mechanical deformation but not to the point of hemorrhage, changes in vessel anatomy and physiology result in decreased perfusability [30]. Following subfailure deformations, cerebral vessels exhibit persistent impaired responses to vasoconstriction and vasodilation stimuli [62], with more dramatic physiological impairments in contraction following larger strain deformation [55, 63]. Deformations in cerebral vessels after axial loading results in tissue softening, perhaps due to ECM reorganization, thus allowing less force to generate equivalent deformation in subsequent injuries [64].

These data suggest an interesting interplay between repetitive diffuse TBIs and neuronal and vascular pathology, however, this study design has several limitations. First, our detailed, unbiased methodology exclusively analyzed the midbrain of pigs. Future studies will need to determine if these trends are conserved across different brain structures, for instance in the cerebral cortex and hippocampus where there were also significant elevations in the incidence of neuronal permeability. Second, vascular changes were most prominent in repetitive TBIs separated by 3 days, however we cannot determine if these changes were due to the time passed since the initial injury or the result of repetitive injury. To answer this question, future studies would need to include a single injury TBI control group survived for 3 days. Third, LY labeling likely tags many neurons with compromised membranes that quickly reseal within minutes of injury. However, this methodology may fail to label neurons which cannot reseal their membranes or are physically damaged without compromising membrane integrity (e.g., neurons or axons exhibiting cytoskeletal discontinuities). In addition, future studies should include brains sectioned in sagittal and axial planes, or a three-dimensional method to better assess vessels that run parallel or oblique to the coronal plane. Finally, these analyses suggest important consequences for neuronal and vessel pathology, however the types of vessels and the consequences of these findings are yet to be determined. Future work characterizing or modeling the extent of tissue oxygenation/ischemia, microthrombi formation, or subfailure vascular deformation in arteries, veins, and capillaries following trauma will help contextualize these findings.

## Conclusion

This study utilized a large animal model of closed-head diffuse TBI to characterize neuropathological and vascular changes across sham, single, and repetitive TBI conditions. Our data suggest that while vascular distribution is not the primary determinant of permeabilized neuron distribution, perivascular proximity plays a significant secondary role in modulating local neuropathological burden. Furthermore, we found exacerbated neuropathology and a reduction in the number of large blood vessels (> 100 μm) following repetitive TBIs separated by 3 days. Together, these analyses suggest that co-occurring changes to neurons and blood vessels following repetitive TBI may generate a unique pathological microenvironment. We believe that better characterization of how repetitive injury timing affects vascular distribution, reactivity, and functionality in relation to pathological distribution will better inform basic scientific questions about TBI biomechanics and cellular responses, while improving our understanding of secondary injury progression and potential targeted therapeutics.

## Supplementary Information

Additional file1 (DOCX 54104 KB)

## Data Availability

The datasets and image analysis methods used within the current study are available from the corresponding author upon reasonable request.
