# Supplementary material for: Spatial associations between neuronal membrane damage and vasculature after repetitive diffuse TBI in pigs
Source: Acta Neuropathol Commun. 2026 May 5;14:137. doi: 10.1186/s40478-026-02309-8 (PMC13312530; doi:10.1186/s40478-026-02309-8)
Supplement: Supplementary file 1 — Additional file1 (DOCX 54104 KB) [file 40478_2026_2309_MOESM1_ESM.docx]

**SUPPLEMENTAL DATA:**

**Supplemental Figure 1. Semi-automated blood vessel analysis pipeline.** (A) Tiled confocal images were thresholded and (B) a binary mask was generated. (C) Voids inside the mask were identified and feature descriptions were collected for each vessel. Each vessel was checked and validated by a blinded technician. (D-H) Composite maps of all animals across injury conditions illustrate blood vessel density and distribution in each injury condition. Within each composite map, colored dots represent the coordinates for each blood vessel from each animal subject within that condition.

**Supplemental Figure 2. Distribution of blood vessel distance to its nearest neighbor.** (A) A histogram representing the distance of a blood vessel to its nearest neighbor from one sham animal. Most blood vessels are within 400µm of its nearest neighbor vessel. (B) Within the 3-day repetitive TBI condition, the distance from each LY^+^ cell to the nearest vessel (C) and the distance from each blood vessel to the nearest LY^+^ cell is reported for each subject.

**Supplemental Figure 3. Closed head rotational injury generates mechanical damage to vessels and neurons.** (A) H&E staining of midbrain tissue after a sham procedure shows healthy cells and vessels distributed throughout the tissue. (B-D) In contrast, following a closed-head rotational TBI, red blood cells associate with vessel walls (arrowheads), bleeding occurs in the intraparenchymal space (arrows), and neurons exhibit hyperpigmentation (asterisk). Scale bars represent 100 µm.

**Supplemental Figure 4. LY dye allows identification of blood vessels in the midbrain.** (A-B) Background fluorescence of LY dye (green) distributed throughout the tissue allowed identification of vessels in the midbrain as black voids. (A-B’) LY-stained tissue was co-labeled with αSMA (red) and CD31 (purple) to confirm that LY^-^ voids were indeed blood vessels. LY^-^ voids colocalized with αSMA and CD31 signal across (C) sham, (D) single, (E) 15-minute repetitive, (F) 3-day repetitive, and (G) 7-day repetitive TBIs conditions. (B’-G’) αSMA and CD31 signal alone from respective images. Scale bars represent (A) 500 µm, (B-B’) 50 µm, and (C-G’) 200 µm.

**Supplemental Figure 5. Large blood vessel density decreases after repetitive TBIs separated by 3 days**. (A) The total number of vessels per animal across injury conditions and (B) the total midbrain area per animal across injury was utilized to calculate the (C) vessel density per animal across injury conditions. The density of (D) small, (E) medium, and (F) large vessels in the porcine midbrains across injury conditions. Note figure A represents data previously reported in Figure 5. These data were presented here again to clearly illustrate how midbrain area and vessel number were utilized to determine vessel density in the midbrain. * denotes significant differences between groups where p<0.05, and ** denotes significant differences between groups where p<0.01.

**Supplemental Figure 6. Neither blood vessels size nor orientation affect LY^+^ cell burden.** (A) A representative grayscale image of a porcine midbrain illustrates the variability of vessel sizes across the tissue. To determine if blood vessel size or aspect ratio affects LY^+^ pathology, we assessed LY^+^ pathology across types of blood vessels after excluding blood vessels that had no evidence of LY^+^ pathology. (B) The median LY^+^ cell number across blood vessel diameters revealed no significant differences across blood vessel size bins. (C) The median LY^+^ cell number across blood vessel aspect ratio revealed no significant differences across blood vessel orientation bins. “ns” denotes no significant differences across groups.
